# Preconception and early-pregnancy risk prediction for birth complications: development of prediction models within a population-based prospective cohort

**DOI:** 10.1186/s12884-022-04497-2

**Published:** 2022-02-28

**Authors:** Rama J. Wahab, Vincent W. V. Jaddoe, David van Klaveren, Marijn J. Vermeulen, Irwin K. M. Reiss, Eric A. P. Steegers, Romy Gaillard

**Affiliations:** 1 The Generation R Study Group, Erasmus MC, University Medical Center, PO Box 2040, 3000 CA Rotterdam, The Netherlands; 2 Department of Pediatrics, Sophia’s Children’s Hospital, Erasmus MC, University Medical Center, Rotterdam, the Netherlands; 3 Department of Public Health, Center for Medical Decision Making, Erasmus MC, University Medical Center, Rotterdam, the Netherlands; 4 Department of Obstetrics & Gynecology, Erasmus University Medical Center, Rotterdam, the Netherlands

**Keywords:** Pregnancy, Preconception, Risk prediction, Birth complications

## Abstract

**Background:**

Suboptimal maternal health already from preconception onwards is strongly linked to an increased risk of birth complications. To enable identification of women at risk of birth complications, we aimed to develop a prediction model for birth complications using maternal preconception socio-demographic, lifestyle, medical history and early-pregnancy clinical characteristics in a general population.

**Methods:**

In a population-based prospective cohort study among 8340 women, we obtained information on 33 maternal characteristics at study enrolment in early-pregnancy. These characteristics covered the preconception period and first half of pregnancy (< 21 weeks gestation). Preterm birth was < 37 weeks gestation. Small-for-gestational-age (SGA) and large-for-gestational-age (LGA) at birth were gestational-age-adjusted birthweight in the lowest or highest decile, respectively. Because of their co-occurrence, preterm birth and SGA were combined into a composite outcome.

**Results:**

The basic preconception model included easy obtainable maternal characteristics in the preconception period including age, ethnicity, parity, body mass index and smoking. This basic preconception model had an area under the receiver operating characteristics curve (AUC) of 0.63 (95% confidence interval (CI) 0.61 to 0.65) and 0.64 (95% CI 0.62 to 0.66) for preterm birth/SGA and LGA, respectively. Further extension to more complex models by adding maternal socio-demographic, lifestyle, medical history and early-pregnancy clinical characteristics led to small, statistically significant improved models. The full model for prediction of preterm birth/SGA had an AUC 0.66 (95% CI 0.64 to 0.67) with a sensitivity of 22% at a 90% specificity. The full model for prediction of LGA had an AUC of 0.67 (95% CI 0.65 to 0.69) with sensitivity of 28% at a 90% specificity. The developed models had a reasonable level of calibration within highly different socio-economic subsets of our population and predictive performance for various secondary maternal, delivery and neonatal complications was better than for primary outcomes.

**Conclusions:**

Prediction of birth complications is limited when using maternal preconception and early-pregnancy characteristics, which can easily be obtained in clinical practice. Further improvement of the developed models and subsequent external validation is needed.

**Supplementary Information:**

The online version contains supplementary material available at 10.1186/s12884-022-04497-2.

## Introduction

Preterm birth, small-for-gestational-age at birth (SGA) and large-for gestational-age at birth (LGA) are among the most common birth complications, affecting a substantial amount of pregnancies [1–5]. These birth complications are not only strongly related to maternal and neonatal morbidity and mortality, but also increase the risk of adverse health outcomes in later life [6–8].

Maternal health and lifestyle during pregnancy are important determinants for fetal development and birth outcomes [9, 10]. Accumulating evidence suggests that preconception and early-pregnancy are crucial periods for the negative effects of adverse maternal lifestyle and physical characteristics on fetal development and birth outcomes [8, 10, 11]. Adverse maternal lifestyle and physical characteristics tend to cluster and are more often present among families with a low socio-economic background. This accumulation of risk factors together further increases the risks of birth complications [12–14]. The preconception period, often defined as 3 months prior to pregnancy, offers an important period for achieving desired health outcomes in preparation for pregnancy [10, 15]. Increasing efforts are made to develop intervention programs to optimize maternal health and lifestyle from preconception onwards to improve birth outcomes [9, 16]. Early identification enables interventions to target those who will benefit most from lifestyle programs and allows intensified antenatal monitoring of those prone to birth complications. Current lifestyle intervention trials, mainly conducted during pregnancy, performed risk selection only on a single maternal characteristic, such as obesity or smoking [17, 18]. To increase the impact of integrated interventions on birth outcomes, screening tools are needed to enable risk stratification of women already from the preconception period within the general population.

Therefore, in a population based prospective cohort study, we aimed to develop prediction models for common birth complications based on maternal preconception socio-demographic, lifestyle, medical history characteristics. We assessed whether maternal early-pregnancy clinical characteristics could further improve model performance. Finally, we translated these models into a clinical prediction tool. Additionally, we examined whether paternal characteristics could improve model performance of the developed prediction models within a subgroup of our population and we examined the predictive performance of the developed prediction models on secondary maternal, delivery and neonatal complications.

## Methods

### Study population

We used data from the Generation R Study, a population-based prospective cohort study from early-pregnancy onwards in Rotterdam, the Netherlands [19]. Inclusion criteria for study enrolment were residency in the study area at delivery date and an expected delivery date from April 2002 until January 2006. For this study, women were included if they were enrolled in the study in first half of pregnancy and had information available on at least one characteristic from the preconception period or early-pregnancy (Supplementary Figure S1). Women with non-singleton pregnancies (*n* = 94) or non-live births were excluded (*n* = 103). After excluding women without information on offspring gestational age or weight at birth (*n* = 84), we had a population for analyses of 8340 women. Information on 6062 fathers was available.

### Candidate predictor variables

We selected maternal preconception and early-pregnancy candidate predictors based on their associations with outcomes in literature and on a priori hypotheses. For selection, we focused on candidate predictors which could be obtained in clinical practice already within the preconception period and during early-pregnancy. Candidate predictors were clustered according to availability in clinical practice and timing of assessment within the cohort. Due to the study enrolment of women from early-pregnancy onwards, maternal preconception characteristics were assessed retrospectively using questionnaires. Early-pregnancy characteristics were assessed using hands on measurements in first half of pregnancy [19]. The result of this literature search is shown in Supplementary Methods S1. Below, assessment of maternal characteristics according to the clustering of the characteristics in our models for analyses is described.

#### Basic preconception characteristics

At study enrolment (median gestational age of 13.9 (95% range 9.9 to 22.9) weeks, we obtained information on maternal age, ethnicity, and parity through questionnaires. Maternal preconception weight and smoking was retrospectively assessed using questionnaires and defined as the weight and smoking just before pregnancy was conceived. Maternal height was measured at study enrolment and pre-pregnancy body mass index (BMI) was calculated.

#### Socio-demographic characteristics

At enrolment, we obtained information on educational level, household income, marital status, current occupational status and planning of pregnancy through questionnaires.

#### Lifestyle characteristics

Information on maternal alcohol consumption, caffeine consumption and multivitamin and folic acid supplementation before pregnancy was obtained through questionnaires [19–22]. We used information about fruit, vegetable and carbohydrate consumption per day and fatty fish consumption per week from a semi quantitative food-frequency questionnaire (FFQ) adapted from the validated FFQ of Klipstein-Grobusch covering the daily food consumption from the previous 3 months [23]. We assessed the overall diet carbohydrate quality at intake by calculating the mean dietary glycemic index [24]. Information on maternal psychological distress was obtained through the Brief Symptom Inventory and psychological distress was defined as a score > 0.71 for the overall psychological symptoms scale [25].

#### Medical history characteristics

Data on the presence of preexisting chronic diseases was assessed using questionnaires at study enrolment and included the presence of chronic bronchitis, severe intestinal disorder, epilepsy, heart conditions, high blood pressure, diabetes, HIV/aids or answering ‘yes’ in the questionnaire on the questionnaire: ‘are you being under treatment at a specialist for a chronic disease’, without further specification for which chronic disease. Information on obstetric complications in previous pregnancies (stillbirth, miscarriage, pre-eclampsia, gestational hypertensive disorders, gestational diabetes, caesarian section, preterm birth, low birth weight, macrosomia), and being a first or second degree relative of the planned biological father was obtained through questionnaires [19]. Information on intra uterine fertilization or intracytoplasmic sperm injection to conceive conception was obtained from medical records [19]. For model development purposes, we combined the presence of any chronic disease into one composite outcome, as well as the history of any obstetric complication.

#### Early-pregnancy clinical characteristics

We measured maternal systolic and diastolic blood pressure in early-pregnancy (median gestational age of 14.4 (95% range 10.9 to 22.1) weeks) using the Omron 907 automated digital oscillometric sphygmomanometer (OMRON Healthcare Europe) [26, 27]. At a median gestational age of 13.2 (95% range 10.5 to 17.2) weeks, we collected venous samples and measured maternal hemoglobin (Hb) concentrations, glucose, total cholesterol, triglycerides, high density lipoprotein cholesterol (HDL-c) and ferritin concentrations using standard laboratory methods [28]. At a median gestational age of 20.4 (95% range 18.8 to 22.9) weeks, we collected venous blood samples and measured 25-hydroxyvitamin D (vitamin D) concentrations and omega-3 fatty acids concentrations [28, 29]. These early-pregnancy clinical characteristics were divided into two clusters: the basic clinical characteristics cluster and the biomarkers cluster.

### Paternal characteristics

Similarly as for the maternal candidate predictors, we selected common paternal characteristics based on a literature search and availability in clinical practice for additional analyses. We obtained information on paternal age, ethnicity, educational level, smoking and alcohol consumption by questionnaire. At enrolment, we measured height, weight and systolic and diastolic blood pressure through one prenatal questionnaire [19]. We calculated paternal BMI.

### Outcomes

#### Primary outcomes

Information about offspring sex, gestational age and weight at birth was obtained from medical records [19]. Preterm birth was defined as gestational age at birth < 37 weeks. Gestational-age and sex-adjusted standard deviation scores for weight at birth were constructed using North European growth standards as the reference growth curve and represent the equivalent of z-scores [30]. Small-for-gestational-age (SGA) and large-for-gestational-age (LGA) at birth were defined as the lowest and highest ten percentiles of gestational age- and sex-adjusted birthweight in the study cohort [30]. As the more extreme ranges of birthweight are even more strongly related to neonatal morbidity and mortality, we additionally tested predictive performance of the developed prediction model on the lowest and highest five percentiles of gestational age- and sex-adjusted birthweight in the study cohort. In our main analyses, we used appropriate size for gestational age at birth as a reference group. Sensitivity analyses using the whole study population as a reference group showed similar results (results not shown). As preterm birth and SGA are strongly correlated and share a majority of risk factors, we combined preterm birth and SGA into one composite outcome variable to increase statistical power [31]. As a sensitivity analysis, we assessed model performance of the developed prediction model for preterm birth and SGA separately. As the etiology of preterm birth may influence results, we performed an additional analysis only including spontaneous preterm births (*n* = 259).

#### Secondary outcomes

We additionally assessed the model performance of the prediction models for the prediction of: 1) maternal complications, including gestational hypertension and preeclampsia; 2) delivery complications, including fetal distress and caesarian section; 3) neonatal complications, including a 5 min Apgar score < 7, low birthweight (< 2500 g) and macrosomia (> 4000 g). Information was obtained from medical records.

### Statistical analyses

Linear variables were used continuously, non-linear variables as quintiles and categorical variables were categorized in common clinical categories (details in Supplementary Table S1). Categorized predictors included one missing category to allow for missing values when using the final risk score in clinical settings. Missing values in linear candidate predictors were imputed using multiple imputations (pooled results of 5 imputed datasets). As a sensitivity analysis, we also performed single imputation of linear continuous candidate predictors using the mean or median, and we observed similar results (results not shown). When using the final models in clinical settings, the mean or median can be inserted in case of a missing linear candidate predictor.

Model estimation was done separately for preterm birth/SGA and LGA. We selected predictors for the models in stages using different multivariable logistic regression models. This approach enables to evaluate whether accurate prediction of birth complications could already be achieved in preconception with a simple model including a low number of easy accessible predictors, or required more complex models including detailed maternal preconception characteristics or additional maternal early-pregnancy biomarker characteristics which can be implemented for potential future routine screening. We defined six models with clustering of variables according to their availability of candidate predictors in clinical settings and timing of assessment (Supplementary Table S1). First, we defined the basic preconception model including maternal age, ethnicity, parity, prepregnancy BMI and smoking. These variables did not require statistical testing prior to inclusion in the model, as they are well known risk factors for birth complications and already routinely available in clincial practice. Second, we extended the basic preconception model by including clusters of maternal candidate predictors ordered by socio-demographic, lifestyle, medical history, early-pregnancy basic clinical and biomarker characteristics. Based on the log-likelihood ratio, we evaluated whether a cluster of variables significantly improved the model and further selected variables from this cluster using backward selection and stopped when all *p*-values were < 0.20 [32]. We included variables of each cluster using an additive approach and tested each cluster of variables in order of above apparence. After variable selection from a cluster, we obtained predicted values from the regression model in one imputed dataset and assessed model performance on discriminative ability, by calculation of the Area Under the Receiver Operating Characteristic Curve (AUC), along with the sensitivity at different false-positive-rates (1-specificity). Results of other imputed datasets were similar (results not shown). We compared AUCs of the basic preconception model with the final model, including the selection of maternal characteristics from all clusters, using DeLong tests [33]. After model estimation, we adjusted effect estimates of the final model for overfitting with an uniform shrinkage factor, calculated using a heuristic formula, and fitted an intercept to the linear predictors of the shrinked effect estimates [34, 35]. To further evaluate performance of the final models for preterm birth/SGA and LGA unadjusted for overfitting, we assessed calibration by dividing our study population in three subsets based on Rotterdam city districts, with a large variation in socio-economic status between subsets (Supplementary Table S2). To assess direction of potential miscalibration, we used calibration plots, which compare the mean of all predicted risks with the mean actual risks. We quantified potential miscalibration using an intercept (calibration-in-the-large). The intercept should ideally be equal to zero and the calibration slope should ideally be equal to one [36]. After development of the final models for prediction of preterm birth/SGA and LGA, we tested whether paternal candidate predictors further improved the model performance for the prediction models for preterm birth/SGA and LGA. We considered this as a separate analysis, as we only had paternal characteristics available in a subgroup of our population and in clinical settings family circumstances may not allow for obtaining characteristics of the biological father. To assess the impact of modifiable preconception characteristics on the predictive performance of the model, we also assessed the AUC of a model only including maternal prepregnancy BMI and smoking as a separate analysis.

Finally, we further examined the clinical applicability of our developed prediction models: 1) based on our developed prediction models, we constructed a risk calculator as a screening tool using the shrinked effect estimates and calculated as an example the risks of delivering a preterm born or SGA newborn and LGA newborn for three fictive women with specific combinations of risk factors; 2) we calculated the number of women with a predicted probability of ≥20% for preterm birth/SGA or ≥ 14% for LGA or higher, who would have actually had a preterm birth/SGA and LGA newborn. Cut offs were determined on the highest quartiles of predicted probabilities within the study population and could potentially serve in clinical settings as a definition of women at increased risk who may require intervention; and 3) we examined the performance of our final prediction models for prediction of secondary maternal, delivery and neonatal complications.

The statistical analyses were performed using the Statistical Package of Social Sciences version 24.0 for Windows (SPSS Inc., Chicago, IL, USA), and R version 3.3.4 (R Foundation for Statistical Computing) (R Core Team 2015).

## Results

### Study population

Table 1 shows the maternal and fetal characteristics according to birth outcomes. Of the 8340 women included, 425 (5.0%) had a newborn born preterm and the mean (SD) birthweight was 3458 (353) grams. Women who had a preterm or SGA newborn were on average more often of non-Dutch or European ethnicity, while women who had a LGA newborn were more often of Dutch or European ethnicity. Maternal prepregnancy BMI was on average higher among women who had a LGA newborn.

**Table 1:** Population characteristics according to birth outcomes (n total population = 8340)

	No adverse birth outcome^**a**^(*n* = 6333)	Preterm birth/small-for-gestational-age^a^ (*n* = 1207)	Large-for-gestational-age^**a**^ (*n* = 834)
**Maternal preconception characteristics**			
Gestational age at enrolment,	13.9 (9.9 to 22.9)	14.1 (8.7 to 23.2)	13.6 (10.2 to 23.2)
Age, mean (SD), years	29.7 (5.2)	28.9 (5.7)	30.9 (4.7)
Ethnicity			
Dutch or European, n (%)	3475 (57.7)	549 (48.5)	536 (67.1)
Surinamese, n (%)	509 (8.4)	178 (15.7)	36 (4.5)
Turkish, n (%)	571 (9.5)	103 (9.1)	60 (7.5)
Moroccan, n (%)	441 (7.3)	49 (4.3)	53 (6.6)
Cape Verdean or Dutch Antilles, n (%)	433 (7.2)	138 (12.2)	38 (4.8)
Other, n (%)	1099 (18.2)	189 (16.7)	131 (16.4)
Body Mass Index, median (95%), kg/m^2^	22.6 (18.0 to 34.9)	22.2 (17.5 to 36.6)	23.8 (19.1 to 38.8)
Parity, n nulliparous (%)	3474 (55.4)	809 (67.9)	338 (41.0)
Education, n higher education (%)	2458 (42.3)	380 (35.0)	404 (52.3)
Smoking, n yes (%)	1477 (26.6)	374 (35.5)	143 (19.6)
Income, n high (%)	2694 (54.9)	3801 (45)	431 (64.8)
Occupational status, employed n (%)	3405 (72.8)	546 (67.8)	496 (78.0)
Planning of pregnancy, n no (%)	1574 (28.3)	380 (36.8)	162 (22.4)
Presence of any chronic disease, n no (%)	1922 (34.7)	390 (32.3)	236 (20.4)
Alcohol consumption, *n* > 1x/week (%)	991 (18.2)	158 (15.5)	146 (20.4)
Vegetable consumption, *n* < 250 g/day (%)	4185 (90.1)	775 (89.8)	566 (87.8)
Fatty fish consumption, *n* < 1x/week, (%)	91 (2.0)	15 (1.7)	1 (0.2)
Caffeine consumption, median (95%), units/day^b^	1.5 (0.0 to 6.0)	1.5 (0.0 to 6.0)	1.5 (0.0 to 6.0)
Multivitamin supplementation, n no (%)	1546 (29.5)	256 (26.7)	152 (35.5)
Mean systolic blood pressure, mean (SD), mmHg	115 (12)	115 (13)	117 (13)
Mean diastolic blood pressure, mean (SD), mmHg	68 (9)	69 (10)	68 (10)
Hb concentrations, mean (SD), mmol/L	7.5 (0.6)	7.5 (0.6)	7.6 (0.6)
Glucose, mean (SD), mmol/L	4.4 (0.8)	4.4 (0.8)	4.6 (0.9)
Triglycerides concentrations, mean (SD), mmol/L	1.36 (0.52)	1.35 (0.51)	1.45 (0.56)
Ferritin concentrations, median (95%), ug/L	52.9 (9.8 to 210.1)	52.2 (9.7 to 212.4)	48.9 (11.2 to 191.5)
Vitamin D concentrations, median (95%), nmol/L	47.5 (7.0 to 122.2)	38.5 (7.0 to 113.1)	31.7 (7.9 to 121.7)
**Birth outcomes**			
Sex, n female (%)	3168 (50.0)	578 (47.9)	410 (49.2)
Gestational age at birth, median (95%), weeks	40.1 (37.4 to 42.4)	39.0 (30.4 to 42.0)	40.3 (35.8 to 42.4)
Birthweight, mean (SD), grams	3458 (353)	2571 (459)	4270 (382)

*Abbreviations: Hb* Hemoglobin

^a^Of large-for-gestational-age newborns, *n* = 34 were preterm, due to which there is overlap between the columns of preterm birth/small-for-gestational-age and large-for-gestational-age

^b^1 unit of caffeine consumption represents the equivalent of 1 cup of coffee (90 mg caffeine)

### Model selection and performance for preterm birth/SGA and LGA

**Fig. 1:**
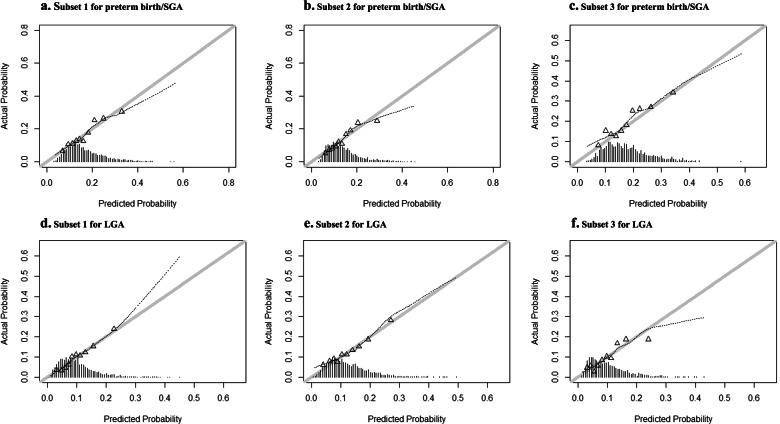
Actual probabilities of preterm birth/SGA and LGA compared with predicted probabilities of preterm birth/SGA and LGA within subsets of the study population based on the final model. Subsets of the populations were constructed based on zip code and reflect subsets with different socio-economic characteristics as shown in Supplementary Table S3. For preterm/SGA was in subset 1 the area under the curve (AUC) 0.65, the intercept − 0.04 and the slope 0.98 (**a**), in subset 2 was the AUC 0.66, the intercept 0.03 and the slope 1.04 (**b**) and in subset 3 was the AUC 0.64, the intercept − 0.03 and the slope 0.90 (**c**). For LGA was in subset 1 the AUC 0.68, the intercept 0.22 and the slope 1.11 (**d**), in subset 2 was the AUC 0.65, the intercept − 0.18 and the slope 0.89 (**e**) and in subset 3 was the AUC 0.68, the intercept − 0.16 and the slope 0.94 (**f**)

For the composite outcome preterm birth/SGA, the basic preconception model had an AUC 0.63 (95% Confidence Interval (CI) 0.61 to 0.65) with a sensitivity of 20% at 90% specificity (Table 2). Maternal characteristics from each cluster improved prediction of the model. Maternal characteristics additionally selected in the model included household income, planning of pregnancy, occupational status, weekly alcohol consumption, weekly fatty fish consumption, daily caffeine consumption, presence of a chronic disease and maternal early-pregnancy diastolic blood pressure, hemoglobin concentrations and vitamin D concentrations. The full prediction model for preterm birth/SGA had an AUC of 0.66 (95% CI 0.64 to 0.67) with a sensitivity of 22% at a 90% specificity, but was significantly improved in comparison to the maternal basic preconception model. A model only including maternal prepregnancy BMI and smoking had an AUC of 0.55 (95% CI 0.54 to 0.57) with a sensitivity of 4% at 90% specificity for the prediction of preterm birth/SGA. When assessing model performance of the full preterm birth/SGA-model on preterm birth and SGA separately, we observed a slightly lower performance for preterm birth and a slightly higher performance for SGA than for the composite outcome preterm birth/SGA (results not shown). Model performances for spontaneous preterm birth/SGA, excluding induced preterm birth, were similar to those as when including both spontaneous and induced preterm birth. The basic preconception model for gestational-age-and-sex-adjusted birthweight within the lowest 5 percentile had an AUC of 0.67 (95% CI 0.64 to 0.70) with a sensitivity of 34% at a 90% specificity, whereas the full model had an AUC of 0.70 (95% CI 0.68 to 0.73), with a sensitivity of 28% at a 90% specificity. The heuristic shrinkage factor was 0.77 for the final model for preterm birth/SGA. Figure 1 shows that the model had a reasonable level of calibration for preterm birth/SGA. Calibration intercepts within subset of the population ranged from − 0.04 to 0.23 and calibration slopes ranged from 0.90 to 1.04. There is an indication that at higher probabilities the model may slightly overestimate cases of preterm birth/SGA in all three subsets of our population.

**Table 2:**
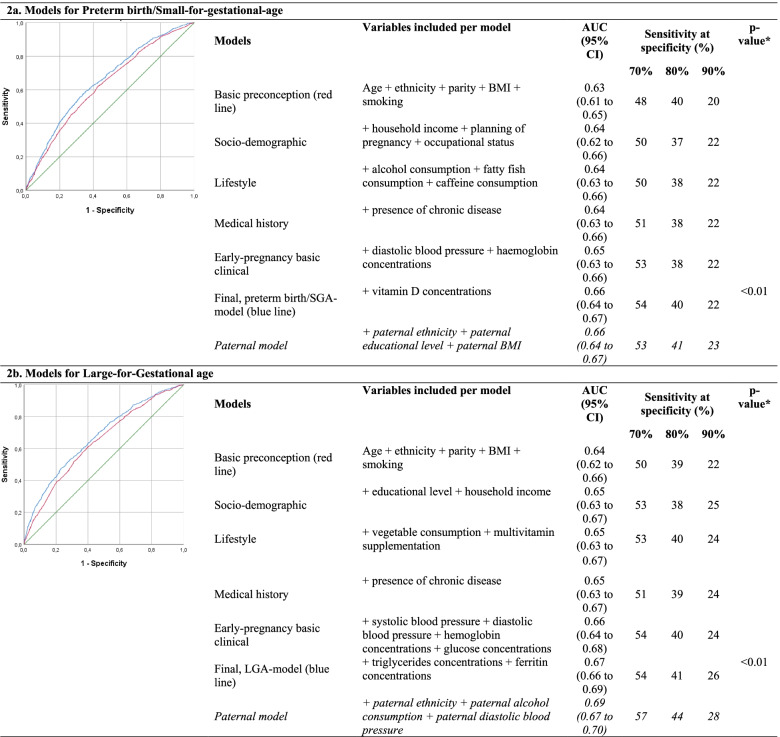
Screening performance for preterm birth/small-for-gestational-age at birth and large-for-gestational-age at birth based on maternal characteristics

*Abbreviations: AUC* area under the Receiver Operating Curve, *CI* confidence interval, *SGA* small-for-gestational-age at birth, *BMI* Body Mass Index

*p-values are obtained using DeLong’s test for comparison of the AUC of the full model with the AUC of the basic preconception model

For LGA, the basic preconception model had an AUC 0.64 (95% CI 0.62 to 0.66) with a sensitivity of 22% at a 90% specificity. Maternal characteristics from each cluster improved prediction of the model. Maternal characteristics additionally selected in the model included educational level, household income, daily vegetable consumption, multivitamin supplementation, presence of a chronic disease and maternal early-pregnancy systolic blood pressure, diastolic blood pressure, hemoglobin concentrations, random glucose concentrations, triglycerides concentrations and ferritin concentrations. The full prediction model for LGA had an AUC of 0.67 (95% CI 0.66 to 0.69), with a sensitivity of 26% at 90% specificity, but was significantly improved in comparison to the basic preconception model (Table 2). A model only including maternal prepregnancy BMI and smoking had an AUC of 0.58 (95% CI 0.56 to 0.60) with a sensitivity of 5% at 90% specificity for the prediction of LGA. The basic preconception model for gestational-age-and-sex-adjusted birthweight within the highest 5 percentile had an AUC of 0.67 (95% CI 0.64 to 0.70), with a sensitivity of 34% at a 90% specificity, whereas the full model had an AUC of 0.70 (95% CI 0.67 to 0.73) with a sensitivity of 31% at a 90% specificity. The heuristic shrinkage factor was 0.76 for LGA. Effect estimates for the final prediction model for LGA before and after shrinkage are shown in Table 3. For LGA, calibration intercepts within subsets of the population ranged from − 0.18 and 0.22 and calibration slopes ranged from 0.89 to 1.11 (Fig. 1). There is an indication that the model may underestimate cases of LGA at higher probabilities in a population consisting of women of non-Dutch or European ethnicity and may overestimate cases of LGA at higher probabilities in a population consisting of women with a lower educational level (Fig. 1).

**Table 3:** Effect estimates in the final models for preterm birth/small-for-gestational-age at birth and for large-for-gestational-age at birth

		Preterm birth/SGA		LGA	
Variable	Categories	Odds Ratio (original)	Odds Ratio (shrunk)	Odds Ratio (original)	Odds Ratio (shrunk)
Intercept		0.04	0.07	0.10	0.11
Age	< 25	0.93	0.95	0.79	0.84
	25–35	*Reference*	*Reference*	*Reference*	*Reference*
	> 35	1.16	1.12	0.83	0.88
Ethnicity	Dutch or European	*Reference*	*Reference*	*Reference*	*Reference*
	Surinamese	1.65	1.44	0.46	0.57
	Turkish	0.86	0.89	0.65	0.73
	Moroccan	0.56	0.65	0.64	0.73
	Cape Verdian or Dutch Antilles	1.51	1.35	0.62	0.71
	Other	0.95	0.96	0.77	0.83
	*Missing*	1.03	1.02	0.63	0.72
Prepregnancy BMI	< 25 kg/m2	*Reference*	*Reference*	*Reference*	*Reference*
	25.0–30.0 kg/m^2^	0.83	0.87	1.61	1.41
	30.1–35.0 kg/m^2^	0.76	0.81	2.09	1.71
	> 35.0 kg/m^2^	0.77	0.83	3.48	2.46
	*Missing*	1.05	1.04	1.44	1.30
Parity	Nulliparity	1.89	1.59	0.57	0.67
	Multiparous	*Reference*	*Reference*	*Reference*	*Reference*
	*Missing*	1.50	1.35	1.15	1.11
Smoking status	No	*Reference*	*Reference*	*Reference*	*Reference*
	Yes	1.32	1.23	0.76	0.82
	*Missing*	0.78	0.83	0.97	0.98
Educational level	Low			1.30	1.21
	High			*Reference*	*Reference*
	*Missing*			0.77	0.82
Household income	Low	1.27	1.19	0.81	0.86
	High	*Reference*	*Reference*	*Reference*	*Reference*
	*Missing*	1.37	1.26	0.91	0.93
Occupational status	Currently employed	*Reference*	*Reference*		
	Applying for a job	0.81	0.86		
	Unemployed and not applying for a job	1.16	1.12		
	*Missing*	1.28	1.20		
Pregnancy planning	Planned pregnancy	*Reference*	*Reference*		
	Unplanned pregnancy	1.11	1.08		
	*Missing*	1.06	1.05		
Alcohol consumption	Never or < 1 drink/week	*Reference*	*Reference*		
	> 1 drinks/week	0.87	0.90		
	*Missing*	1.07	1.05		
Vegetable intake	< 250 g/day			0.69	0.77
	≥250 g/day			*Reference*	*Reference*
	*Missing*			0.65	0.73
Weekly fatty fish consumption	<1x/week	1.15	1.11		
	1-2x/week	*Reference*	*Reference*		
	>2x/week	2.59	2.01		
	*Missing*	1.05	1.04		
Daily caffeine consumption	*< 2/day*	*Reference*	*Reference*		
	≥2/day	1.14	1.10		
	*Missing*	0.86	0.90		
Multivitamin supplementation	Yes			*Reference*	*Reference*
	No			1.26	1.18
	*Missing*			1.27	1.19
History of a chronic disease	No	*Reference*	*Reference*	*Reference*	*Reference*
	Yes	1.13	1.10	0.85	0.89
	*Missing*	1.37	1.26	0.86	0.90
Systolic blood pressure	Per 10 mmHg increase			1.08	1.06
Diastolic blood pressure	Per 10 mmHg increase	1.10	1.07	0.92	0.94
Hb concentrations	First quintile (3.9 to 7.0 mmol/l)	1.17	1.12	1.15	1.11
	Second quintile (7.1 to 7.4 mmol/l)	1.10	1.07	0.97	0.98
	Third quintile (7.5 to 7.6 mmol/l)	*Reference*	*Reference*	*Reference*	*Reference*
	Fourth quintile (7.7 to 8.0 mmol/l)	1.15	1.11	1.25	1.18
	Fifth quintile (8.10 to 11.30 mmol/l)	1.33	1.23	0.92	0.94
	*Missing*	1.25	1.17	1.09	1.06
Random glucose concentrations	Per mmol/L increase			1.16	1.12
Triglyceride concentrations	Per mmol/L increase			1.18	1.13
Ferritine concentrations	First quintile (1.5 to 26.4 μg/l)			1.18	1.13
	Second quintile (26.4 to 42.5 μg/l)			0.87	0.91
	Third quintile (42.5 to 62.8 μg/l)			*Reference*	*Reference*
	Fourth quintile (62.8 to 95.8 μg/l)			0.69	0.77
	Fifth quintile (95.9 to 390.4 μg/l)			0.86	0.89
	*Missing*			0.87	0.91
Vitamin D concentrations	Per 10 nmol/l increase	0.94	0.96		

### Paternal analyses

Paternal baseline characteristics are shown in Supplementary Table S3. Paternal characteristics additionally selected for the full model for preterm birth/SGA included paternal ethnicity, paternal educational level and paternal BMI, but these variables did not improve the final model performance (AUC of 0.66 (95% CI 0.64 to 0.67) with a sensitivity of 23% at 90% specificity) (Table 2). Paternal characteristics additionally selected for the full model for LGA included paternal ethnicity, paternal alcohol consumption and paternal diastolic blood pressure and only slightly improved the model performance (AUC of 0.69 (95% CI 0.67 to 0.70) with a sensitivity of 28% at 90% specificity).

### Clinical applicability of the maternal prediction models

**Fig. 2:**
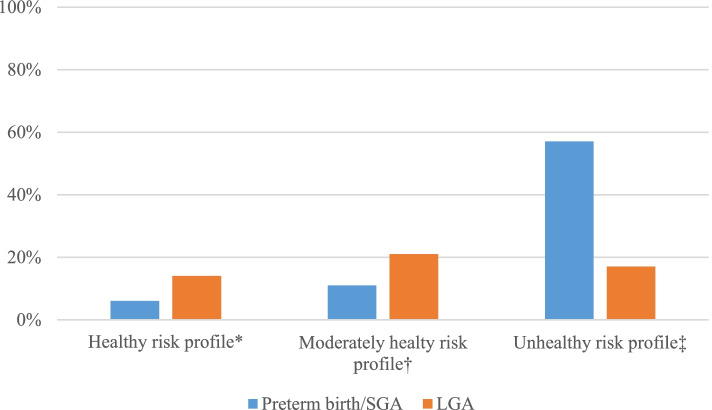
Predicted risks for examples of women at low-, normal- and high risk of an adverse birth outcome obtained from the risk calculator. Values are percentages for the risk of having an adverse birth outcome based on the risk calculator developed from the final prediction models for preterm birth/SGA and LGA. *Healthy risk profile represents a women with age 27, Turkish, BMI 20 kg/m^2^, multiparous, non-smoker, high educated, high household income, employed, planned pregnancy, alcohol consumption none, vegetable intake 300 g/day, fatty fish consumption 1-2x/week, caffeine consumption 1 cup/day, multivitamin supplementation yes, chronic disease no, systolic blood pressure 110 mmHg, diastolic blood pressure 70 mmHg, Hb 7.5 mmol/L, glucose concentrations 3.0 mmol/L, triglyceride concentrations 2.0 mmol/L, ferritin concentrations 50 μg/L and vitamin D concentrations 100 nmol/L . †Moderately healthy risk profile represents a women with age 26, Dutch, BMI 27 kg/m^2^, nulliparous, smoking no, high educated, high household income, employed, planned pregnancy, alcohol consumption 3 glasses/week, vegetable consumption 300 g/day, fatty fish consumption 1x/week, caffeine consumption none, multivitamin supplementation no, history of chronic disease yes, systolic blood pressure 120 mmHg, diastolic blood pressure 80 mmHg, Hb 7.2 mmol/L, glucose concentrations 4.0 mmol/L, triglyceride concentrations 3.0 mmol/L, ferritin concentrations 50 μg/L and vitamin D concentrations 60 nmol/L. ‡Unhealthy risk profile represents a women age 36, Surinamese, BMI 38 kg/m^2^, nulliparous, smoking yes, low educated, low household income, unemployed and not applying for a job, unplanned pregnancy, alcohol consumption less than 1 drink/day, vegetable intake 100 g/day, fatty fish consumption >2x/week fatty fish, caffeine consumption 5 cups/day, multivitamin supplementation no, history of chronic disease yes, systolic blood pressure 140 mmHg, diastolic blood pressure 95 mmHg, Hb 10.0 mmol/L, glucose concentrations 7.0 mmol/L, triglyceride concentrations 4 mmol/L, ferritin concentrations 100 μg/L and vitamin D concentrations 10 nmol/L

**Fig. 3:**
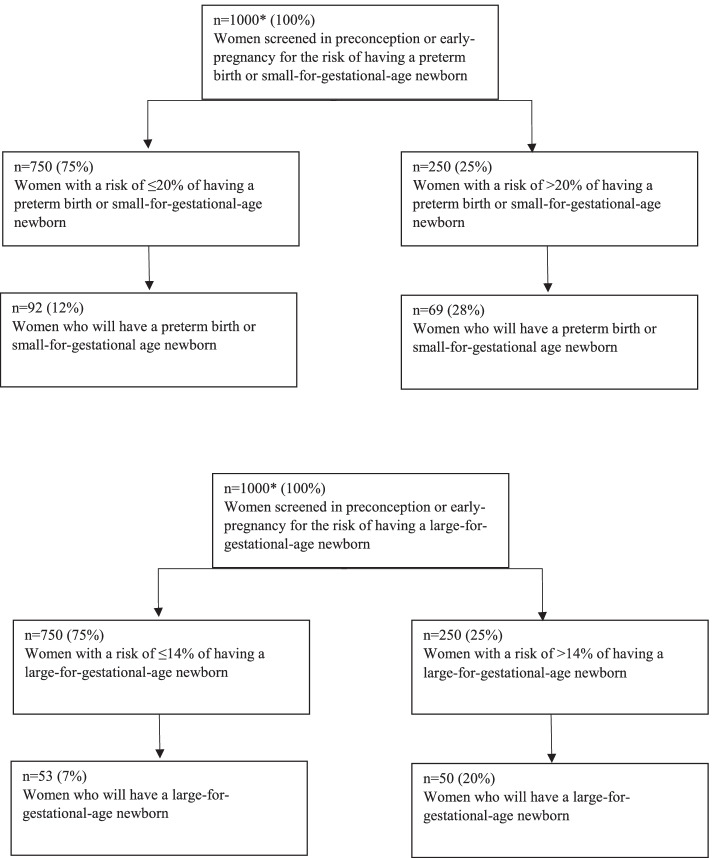
Frameworks of women with an increased risk of preterm birth/small-for-gestational age and Large-for-gestational-age newborns. *For interpretation of this framework, we choose a random number of 1000 women to illustrate how proportions of this framework translate to numbers within an actual population

To illustrate clinical translation of the developed maternal full prediction models, we calculated the risks of preterm birth/SGA or LGA for examples of three women with different risk profiles ranging from healthy to unhealthy in Fig. 2, using the risk calculator as screening tool (Supplementary Material, Excel sheet 1). For a woman with a healthy risk profile the risk of delivering a preterm/SGA and LGA newborn was 5 and 14%, respectively. For a woman with an unhealthy risk profile the risk was 56 and 17% for preterm/SGA and LGA newborn, respectively. Figure 3 shows that of women with a risk > 20% for having a preterm birth/SGA newborn, 27% would have actually had a preterm birth/SGA newborn. Of women with a risk of > 14% for having a LGA newborn, 20% would have actually had a LGA newborn.

The full maternal prediction model for preterm birth/SGA had AUCs ranging from 0.68 (95% CI 0.66 to 0.70) to 0.80 (95% CI 0.78 to 0.83) for the outcomes gestational hypertension, preeclampsia, fetal distress, low Apgar score, low birthweight and macrosomia (Table 4). Model performance for caesarean delivering was slightly lower. Secondary model performance for the prediction of maternal pregnancy complications, delivery complications and neonatal complications by the developed full maternal prediction model for LGA was largely similar (Table 4). Model performance of the basic preconception model for the prediction of secondary outcomes is shown in Supplementary Table S4 and was poorer in comparison to the full maternal prediction models for all secondary outcomes.

**Table 4:** Model performance for maternal, delivery and neonatal complications

Models	AUC (95% CI)	Sensitivity at specificity (%)		
		70%	80%	90%
**Maternal pregnancy complications**				
** Gestational hypertension**				
Preterm birth/SGA-model^a^	0.80 (0.78 to 0.83)	75	63	45
LGA-model^b^	0.81 (0.79 to 0.83)	77	63	44
** Pre-eclampsia**				
Preterm birth/SGA-model^a^	0.77 (0.74 to 0.80)	70	58	33
LGA-model^b^	0.77 (0.74 to 0.81)	72	59	40
**Delivery complications**				
** Fetal distress**				
Preterm birth/SGA-model^a^	0.68 (0.66 to 0.70)	53	40	25
LGA-model^b^	0.68 (0.66 to 0.70)	55	41	23
** Caesarian section**				
Preterm birth/SGA-model^a^	0.65 (0.64 to 0.67)	50	37	23
LGA-model^b^	0.66 (0.64 to 0.67)	51	39	24
**Neonatal complications**				
** Low Apgar**				
Preterm birth/SGA-model^a^	0.70 (0.65 to 0.76)	60	50	36
LGA-model^b^	0.72 (0.66 to 0.77)	65	48	35
** Low birthweight**				
Preterm birth/SGA-model^a^	0.70 (0.67 to 0.73)	59	45	29
LGA-model^b^	0.70 (0.67 to 0.72)	59	44	27
** Macrosomia**				
Preterm birth/SGA-model^a^	0.71 (0.67 to 0.74)	61	50	34
LGA-model^b^	0.71 (0.67 to 0.75)	62	54	35

^a^Final model with variables selected on preterm birth/SGA include maternal age, ethnicity, parity, Body Mass Index, smoking, household income, planning of pregnancy, occupational status, weekly alcohol consumption, weekly fatty fish consumption, daily caffeine consumption, presence of chronic disease and maternal early-pregnancy diastolic blood pressure, hemoglobin concentrations and vitamin D concentrations

^b^Final model with variables selected on LGA include maternal age, ethnicity, parity, Body Mass Index, smoking, educational level, household income, daily vegetable consumption, presence of chronic disease, multivitamin supplementation and maternal early-pregnancy systolic blood pressure, diastolic blood pressure, hemoglobin concentrations, glucose concentrations, triglyceride concentrations and ferritin concentrations

## Discussion

In this population-based prospective cohort study, we observed that easy obtainable maternal characteristics that can be applied in the preconception period and early-pregnancy can already predict common birth complications moderately. Addition of detailed maternal preconception and early-pregnancy characteristics led to a significant, though small, improvement of the developed prediction models. These final maternal prediction models developed based on preterm birth, SGA and LGA had a better performance for the prediction of secondary maternal, delivery and neonatal complications than for the prediction of preterm birth, SGA and LGA. Paternal characteristics did not strongly improve prediction of common birth complications, in addition to these maternal characteristics.

### Methodological considerations

The major strength of this study is the prospective data collection and information on a large number of maternal characteristics. We considered clinical applicability in the development of the prediction models by using a stepwise model estimation including maternal preconception socio-demographic, lifestyle, medical history characteristics and early-pregnancy clinical characteristics. The response rate at birth was 61%. The non-response at baseline and the relatively low prevalence of birth complications in our sample might have let to selection towards a relatively healthy population. Although we corrected for overfitting in our models to maintain adequate prediction performance in new populations and the models had good calibration in subsets of our population with large in-between differences in socio-demographic characteristics, external validation of the prediction models is needed to assess generalizability to other populations. We obtained high quality data for a large number of maternal characteristics through hands-on measurements, blood withdrawal and questionnaires. Although we used validated questionnaires to assess socio-demographic and lifestyle characteristics, these measurements may still have been affected by measurement error, recall bias and reporting bias. Also, we were only able to create a composite predictor for the presence of chronic diseases and a history of obstetric complications. We had a limited proportion of women with chronic diseases or obstetric complications in medical history available, due to our study sample with a relatively high proportion of nulliparous women. All included chronic diseases and complications in previous pregnancies are associated with either preterm birth, SGA or LGA. We therefore do not consider the use of composite candidate predictors to have affected the overall model performance. To assess the predictive value on birth complications of each chronic disease and history of obstetric complications in previous pregnancies separately, future studies with higher proportions of women with diseases and multiparous women should use further differentiation for these candidate predictors. We only had information on maternal biomarker characteristics available from non-fasting samples in the first half of pregnancy. Previous studies have shown that maternal early-pregnancy biomarkers at least partly reflect maternal biomarkers in the preconception period [37, 38]. However, to enable use of the full model already before pregnancy and in earlier stages of pregnancy, further studies are needed to replicate findings using maternal biomarker characteristics already obtained prior or in earlier stages of pregnancy.

### Interpretation of main findings

Increasing efforts are made to develop integrated strategies to optimize maternal health and lifestyle already from preconception onwards to improve pregnancy outcomes [10, 39–42]. To identify women who will most likely benefit from lifestyle intervention programs, tools are urgently needed to advance screening for common birth complications already from the moment when a woman or couple are planning a pregnancy onwards’ [43].

Only a few previous studies developed models for prediction of birth complications [17, 18, 44]. These studies focused on pregnancy, but not the preconception period, and used selected populations, such as nulliparous women or women with a low socio-economic status. A study among 5606 nulliparous pregnant women developed a prediction model for SGA using maternal birthweight, gestational weight gain, biomarkers and ultrasound characteristics at 20 weeks gestation and observed an AUC of 0.69 [17]. In the same cohort, predictive performance for LGA at birth based on the same characteristics at 20 weeks gestation was similar [18]. In a study among 263 deprived pregnant women, a prediction model using maternal socio-demographic characteristics obtained during pregnancy for the prediction of the composite outcome preterm birth, low birthweight, intrauterine fetal demise, or neonatal death was developed with an AUC of 0.79 in the training set, but an AUC of 0.63 in the validation set [44].

We developed two prediction models for preterm birth or SGA and LGA and translated these models in an online risk calculator as screening tool, which can provide an extensive maternal risk profile already within the preconception period and in early pregnancy. As preterm birth, SGA and LGA are major risk factors for maternal and neonatal mortality and morbidity, it is necessary to consider this full range of birth complications in maternal risk stratification. Model performances for only spontaneous preterm birth as an outcome, were similar to those when also including induced preterm birth. Importantly, we observed a better model performance of the developed prediction models for the detection of secondary maternal, delivery and neonatal complications and of newborns within the more extreme ranges of abnormal birthweight. Our range of birthweight may cover SGA or LGA newborns that are rather constitutionally than pathologically small or large for their gestational age [45]. Birthweight in the extreme ranges may reflect pathologically SGA or LGA newborns which have higher risks of morbidity and mortality, than newborns who are constitutionally small or large for their gestational age [45]. Although we aimed to predict birth complications without assumptions on etiology or causality, our findings suggest that our developed prediction models may be more sufficient for identification of women at increased risks of related pregnancy complications or birth complications that reflect more pathological pathways than for the prediction of newborns being constitutionally small or large for their gestational age. We developed our models in a population-based cohort study using an extensive scope of detailed maternal characteristics clustered based on clinical availability and timing of assessment. We already observed a moderate screening performance for only a small group of maternal preconception characteristics, which can easily and routinely be obtained in clinical practice. We assessed whether the model improved by addition of maternal socio-demographic, lifestyle, medical history and early-pregnancy characteristics using the likelihood ratio test and the DeLong test. What can be considered as clinical relevant improvement depends on professional judgement. Although models improved significantly based on the statistical tests, differences in predictive performance of the prediction models were relatively small after the addition of more detailed maternal preconception and early-pregnancy clinical characteristics. This may demonstrate the importance of the maternal age, ethnicity, prepregnancy BMI, parity and smoking in clinical risk prediction. However, as our study population was a relatively healthy population, the range of the other more detailed maternal characteristics in our sample may also reflect a relatively healthy range. Possibly, the predictive value of more detailed maternal characteristics is stronger at the more extreme levels of these characteristics. Paternal characteristics did not strongly improve risk prediction in addition to maternal characteristics. Thus, our findings suggest that early risk prediction of common birth complications is moderate, when using maternal characteristics which can already be obtained in clinical practice the preconception period and in early-pregnancy. Due to the modest performance of our models and lack of external validation, applicability of our models as a screening tool in clinical practice is limited. Further improvement of models is needed, by for example using novel biomarkers or by additionally providing integrated stratification within models to enable applicability to particular settings or subgroups. Subsequently, external model validation is needed to assess reproducibility and transportability to other populations prior to implementation of models as a screening tool in clinical practice.

### Clinical implications

Risk assessment based on clinically available characteristics in preconception and early-pregnancy remains a major challenge. Additional information throughout pregnancy, such as fetal and placental ultrasound, enable more accurate risk prediction for birth complications [46, 47]. However, risk prediction in this time window is often too late for individuals to achieve desired health outcomes to prevent birth complications [48, 49]*.* Previous intervention studies often included women based only on one risk factor such as overweight or smoking [17, 18]. However, our results suggest that risk stratification based only on these maternal modifiable characteristics is not sufficient for the prediction of birth complications. Our more extensive prediction models, enabling early prediction of birth complications, may therefore serve as a first step in future risk stratification. Although our models have a modest performance, early selection in preconception and early-pregnancy of women who can be considered to be at increased risk of pregnancy complications, may be useful for the development of effective and timely integrated interventions for improving birth outcomes. Development of tailored interventions targeting modifiable maternal characteristics is needed to prevent neonatal morbidity and mortality and to improve long-term maternal and offspring health [42, 50, 51]. Further randomized controlled trials are needed to assess whether our models may aid in the early-risk stratification for the development of timely and effective interventions in women already from preconception onwards. After development of effective interventions, optimal screening methods need to be developed to enable screening for women at increased risk of birth complications from preconception onwards on a population level.

## Conclusions

Maternal characteristics, which can easily be obtained in clinical practice already during the preconception period and in early-pregnancy, can aid in the early prediction of common birth complications within the general population. Further improvement of the developed models and subsequent external validation is needed to enable identification of women from preconception onwards at risk of birth complications in future intervention studies or clinical practice.

## Supplementary Information

**Additional file 1.**

## Data Availability

The datasets generated during and/or analyzed during the current study are not publicly available due to regulations on sensitive personal data but are available from the corresponding author on reasonable request.
