# Supplementary material for: Preconception and early-pregnancy risk prediction for birth complications: development of prediction models within a population-based prospective cohort
Source: BMC Pregnancy Childbirth. 2022 Feb 28;22:165. doi: 10.1186/s12884-022-04497-2 (PMC8886786; doi:10.1186/s12884-022-04497-2)
Supplement: Supplementary file 1 — Additional file 1. [file 12884_2022_4497_MOESM1_ESM.docx]

**Supplementary material**

**Preconception and early-pregnancy risk prediction for birth complications: development of prediction models within a population-based prospective cohort** *Running title: Risk prediction of birth complications*

Rama J. Wahab, PhD student^1,2^, Vincent W.V. Jaddoe, professor of pediatrics^1,2^, David van Klaveren, assistant professor of statistics and medical decision making^3^, Marijn J. Vermeulen, assistant professor of pediatrics^1,2^, Irwin K.M. Reiss, professor of pediatrics^2^, Eric A.P. Steegers, professor of obstetrics and gynecology^4^, Romy Gaillard MD PhD, assistant professor of pediatrics^1,2^

1. The Generation R Study Group, Erasmus MC, University Medical Center, Rotterdam, the Netherlands.
2. Department of Pediatrics, Sophia’s Children’s Hospital, Erasmus MC, University Medical Center, Rotterdam, the Netherlands.
3. Department of Public Health, Center for Medical Decision Making, Erasmus MC, University Medical Center, Rotterdam, the Netherlands
4. Department of Obstetrics & Gynecology, Erasmus University Medical Center, Rotterdam, the Netherlands

**Content**

Figure S1 Flow chart of the study participants

Methods S1 Literature search

Table S1 Categories of candidate predictors per model

Table S2 Baseline characteristics not included in the models

Table S3 Socio-demographic characteristics of subsets for calibration

Table S4 Paternal characteristics

Table S5 Model performance of the core model for secondary outcomes

**Supplementary Figure S1. Flow chart of the study participants**

n= 8663
Mothers enrolled during early-pregnancy

n=323
Twin pregnancy n= 94

Induced abortion n= 29

Intrauterine fetal death n= 74

Loss to follow up n= 42

No information on gestational

age or weight at birth n= 84

n= 8340
Women with singleton live births with information available on at least one characteristic in preconception or early-pregnancy

**Supplementary Methods S1. Literature search**

((“pregnancy” [mesh]) or pregnancy or (preconception) or (prenatal))

AND (“risk factors” [Mesh] OR “Life Style” [mesh] or “health behavior” [mesh] and (risk factor*) or (life style) or (diet*))

AND (“premature birth” [Mesh] or “Infant, Low Birth Weight” [mesh] or “Pregnancy Complications” [mesh] or (pregnancy outcome) or (birth) or (growth restriction) OR (low birth weight) or macrosom*)

AND ((meta-analysis [Publication Type] OR review [Publication Type] OR systematic review [Publication Type]) AND (english[Language]) AND (("2010"[Date - Publication] : "3000"[Date - Publication])))

**Supplementary Table S1. Categories of candidate predictors**

|  | Variable | Categories |
| --- | --- | --- |
| *Basic preconception characteristics* | Age | <25 |
|  |  | 25-35 |
|  |  | >35 |
|  | Ethnicity | Dutch or European |
|  |  | Surinamese |
|  |  | Turkish |
|  |  | Moroccan |
|  |  | Cape Verdian or Dutch Antilles |
|  |  | Other |
|  |  | *Missing* |
|  | Parity | Nulliparity |
|  |  | Multiparous |
|  |  | *Missing* |
|  | Prepregnancy BMI | <25.0 kg/m^2^ |
|  |  | 25.0; 29.9.0 kg/m^2^ |
|  |  | 30.0; 35.0 kg/m^2^ |
|  |  | >35.0 kg/m^2^ |
|  |  | *Missing* |
|  | Smoking | No |
|  |  | Yes |
|  |  | *Missing* |
| *Socio-demographic characteristics* | Education | Low |
|  |  | High |
|  |  | *Missing* |
|  | Income | Low |
|  |  | High |
|  |  | *Missing* |
|  | Marital status | Married/living together |
|  |  | No partner/unstable relationship |
|  |  | *Missing* |
|  | Occupation | Currently employed |
|  |  | Applying for a job |
|  |  | Unemployed and not applying for a job |
|  |  | *Missing* |
|  | Pregnancy planning | Planned pregnancy |
|  |  | Unplanned pregnancy |
|  |  | *Missing* |
| *Lifestyle characteristics* | Alcohol consumption | Never or ≤ 1 drink/week |
|  |  | >1 drink/week |
|  |  | *Missing* |
|  | Multivitamin supplementation | Yes |
|  |  | No |
|  |  | *Missing* |
|  | Folic acid supplementation | yes |
|  |  | No |
|  |  | *Missing* |
|  | Caffeine consumption | *<2/day* |
|  |  | ≥2/day |
|  |  | *Missing* |
|  | Fruit consumption | ≥200 grams/day |
|  |  | <200 grams/day |
|  |  | *Missing* |
|  | Vegetable consumption | ≥250 grams/day |
|  |  | <250 grams/day |
|  |  | *Missing* |
|  | Fatty fish consumption | <1x/week |
|  |  | 1-2x/week |
|  |  | >2x/week |
|  |  | *Missing* |
|  | Dietary glycemic index | per 1 increase |
|  | Stress | BSI score ≤0.71 |
|  |  | BSI score >0.71 |
|  |  | *Missing* |
| *Medical history characteristics* | History of a chronic disease | No |
|  |  | Yes |
|  |  | *Missing* |
|  | Obstetric complications in history | No |
|  |  | Yes |
|  |  | *Missing* |
|  | IVF or ICSI pregnancy | No |
|  |  | Yes |
|  |  | *Missing* |
|  | Consanguinity with planned biological father | No |
|  |  | Yes |
|  |  | *Missing* |
| *Early-pregnancy clinical characteristics* | Systolic blood pressure | per 10 mmHg increase |
|  | Diastolic blood pressure | per 10 mmHg increase |
|  | Hb | First quintile (3.9; 7.0 mmol/l) |
|  |  | Second quintile (7.1; 7.4 mmol/l) |
|  |  | Third quintile (7.5; 7.6 mmol/l) |
|  |  | Fourth quintile (7.7; 8.0 mmol/l) |
|  |  | Fifth quintile (8.10; 11.30 mmol/l) |
|  |  | *Missing* |
|  | Random Glucose | per 1 mmol/L increase |
|  | Total cholesterol | Per 1 mmol/L increase |
|  | HDL | per 1 mmol/L increase |
|  | Triglycerides | per 1 mmol/L increase |
|  | Ferritine | First quintile (1.5; 26.4 ug/l) |
|  |  | Second quintile (26.4; 42.5 ug/l) |
|  |  | Third quintile (42.5; 62.8 ug/l) |
|  |  | Fourth quintile (62.8; 95.8 ug/l) |
|  |  | Fifth quintile (95.9; 390.4 ug/l) |
|  |  | *Missing* |
|  | Vitamin D | per 10 nmol/l increase |
|  | Omega-3 fatty acids | per 10 mg/l increase |
| *Paternal characteristics* | Age | <25 |
|  |  | 25-35 |
|  |  | >35 |
|  | Ethnicity | Dutch or European |
|  |  | Surinamese |
|  |  | Turkish |
|  |  | Moroccan |
|  |  | Cape Verdian or Dutch Antilles |
|  |  | Other |
|  |  | *Missing* |
|  | Prepregnancy BMI | <25.0 kg/m^2^ |
|  |  | 25.0; 29.9.0 kg/m^2^ |
|  |  | >30 kg/m^2^ |
|  |  | *Missing* |
|  | Smoking | No |
|  |  | Yes |
|  |  | *Missing* |
|  | Alcohol consumption in past two months | No |
|  |  | Yes |
|  |  | *Missing* |
|  | Systolic blood pressure | per 10 mmHg increase |
|  | Diastolic blood pressure | per 10 mmHg increase |

Green represents the reference category, grey represents the missing category.

**Supplementary Table S2. Characteristics not selected into prediction models according to birth outcomes**

|  | **No adverse birth outcome*** (n=6333) | **Preterm birth/small-for-gestational-age*** (n=1207) | **Large-for-gestational-age*** (n=834) |
| --- | --- | --- | --- |
| **Maternal preconception characteristics** |  |  |  |
| Marital status, n no partner/stable relation (%) | 789 (13.6) | 244 (22.5) | 65 (8.5) |
| History of any obstetric complication, n yes (%)† | 2027 (95.0) | 390 (97.3) | 258 (95.2) |
| IVF/ICSI pregnancy, n yes (%) | 45 (0.7) | 9 (0.9) | 6 (0.8) |
| Consanguinity with biological father, n yes (%) | 225 (4.1) | 33 (3.2) | 30 (4.1) |
| Fruit consumption, n <200grams/day (%) | 2970 (63.9) | 590 (68.4) | 390 (60.5) |
| Daily dietary glycemic index, mean (SD) | 58.2 (3.4) | 58.5 (3.6) | 57.9 (3.2) |
| Folic acid supplementation, n no (%) | 1343 (28.4) | 312 (35.9) | 140 (22.2) |
| Psychological distress, n yes (%) | 510 (10.4) | 120 (13.5) | 57 (8.7) |
| Total cholesterol concentrations, mean (SD), mmol/L | 4.81 (0.85) | 4.80 (0.88) | 4.82 (0.89) |
| HDL-concentrations, mean (SD), mmol/L | 1.77 (0.35) | 1.77 (0.35) | 1.73 (0.35) |
| Omega-3 fatty acids concentrations, mean (SD), mg/L | 104.1 (27.7) | 100.6 (26.9)  (26.1) | 106.2 (26.1) |

*Of large-for-gestational-age newborns, n=34 were preterm, due to which there is overlap between the columns of preterm birth/small-for-gestational-age and large-for-gestational-age

**Supplementary Table S3. Socio-demographic characteristics of subsets for calibration**

|  | **Subset 1** (n=4091) | **Subset 2** (n=3035) | **Subset 3** (n=1214) |
| --- | --- | --- | --- |
| **Maternal socio-demographic characteristics** |  |  |  |
| Ethnicity |  |  |  |
| Dutch or European, n (%) | 1772 (43.3) | 2215 (73.0) | 553 (45.6) |
| Surinamese, n (%) | 403 (9.9) | 196 (6.5) | 123 (10.1) |
| Turkish, n (%) | 506 (12.4) | 110 (3.6) | 116 (9.6) |
| Moroccan, n (%) | 369 (9.0) | 102 (3.4) | 69 (5.7) |
| Cape Verdean or Dutch Antilles, n (%) | 364 (8.9) | 98 (3.2) | 145 (11.9) |
| Other, n (%) | 702 (17.2) | 495 (16.3) | 217 (17.9) |
| Education, n higher education (%) | 1939 (47.4) | 1644 (54.2) | 346 (28.5) |
| Income, n high (%) | 1633 (39.9) | 720 (23.7) | 561 (46.2) |
| Occupational status, employed n (%) | 1924 (47.0) | 2016 (66.4) | 492 (40.5) |
| Marital status, n no partner/stable relation (%) | 468 (11.4) | 150 (4.9) | 111 (9.1) |
| Planned pregnancy, n no (%) | 1162 (28.4) | 562 (18.5) | 388 (32.0) |
| **Birth outcomes** |  |  |  |
| Preterm birth | 217 (5.3) | 128 (4.2) | 80 (6.6) |
| Small-for-gestational-age | 436 (10.7) | 254 (8.4) | 144 (11.9) |
| Large-for-gestational-age | 370 (9.0) | 356 (11.7) | 108 (8.9) |

**Supplementary Table S4. Paternal characteristics** *(n=6062)*

|  | **No adverse birth outcome*** (n=4631) | **Preterm birth***(n=285) | **Small-for-gestational-age*** (n=545) | **Large-for-gestational-age*** (n=601) |
| --- | --- | --- | --- | --- |
| **Paternal characteristics** |  |  |  |  |
| Age, mean (SD), years | 32.8 (5.7) | 32.2 (6.1) | 31.8 (6.0) | 33.4 (5.1) |
| Ethnicity |  |  |  |  |
| Dutch or European, n (%) | 2711 (58.5) | 155 (54.4) | 248 (45.5) | 431 (71.7) |
| Surinamese, n (%) | 277 (6.0) | 23 (8.1) | 73 (13.4) | 12 (2.0) |
| Turkish, n (%) | 311 (6.7) | 21 (7.4) | 37 (6.8) | 27 (4.5) |
| Moroccan, n (%) | 185 (4.0) | 9 (3.2) | 26 (4.8) | 19 (3.2) |
| Cape Verdean or Dutch Antilles, n (%) | 235 (5.3) | 16 (5.6) | 45 (8.2) | 17 (2.8) |
| Other, n (%) | 395 (9.0) | 24 (9.0) | 46 (9.0) | 44 (7.6) |
| Body Mass Index, mean (SD), kg/m^2^ | 25.3 (3.5) | 25.3 (3.5) | 24.9 (3.5) | 25.6 (3.5) |
| Education, n. higher education (%) | 1985 (42.9) | 105 (36.8) | 168 (30.8) | 316 (52.6) |
| Smoking, n yes (%) | 1653 (35.7) | 106 (37.2) | 216 (39.6) | 202 (33.6) |
| Alcohol consumption in past 2 months, n yes (%) | 3173 (68.5) | 176 (61.8) | 353 (64.8) | 451 (75.0) |
| Mean systolic blood pressure, mean (SD), mmHg | 130 (14) | 130 (13) | 129 (13) | 130 (13) |
| Mean diastolic blood pressure, mean (SD), mmHg | 73 (11) | 73 (12) | 72 (11) | 73 (10) |

**Supplementary Table S5. Model performance for the core model for secondary outcomes**

| **Maternal pregnancy complications and adverse birth outcomes** | | | | |
| --- | --- | --- | --- | --- |
| **4a. Model performance for gestational hypertension and preeclampsia (n total population=8136, ncases=495)** | | | | |
| **Models** | **AUC (95% CI)** | **Sensitivity at specificity (%)** | | |
|  |  | **70%** | **80%** | **90%** |
| Core^a^ | 0.70 (0.67; 0.72) | 60 | 43 | 28 |
| **4b. Fetal distress (n total population=8030, ncases=598)** |  | | | |
| **Models** | **AUC (95% CI)** | **Sensitivity at specificity (%)** | | |
|  |  | **70%** | **80%** | **90%** |
| Core^a^ | 0.70 (0.67; 0.72) | 60 | 43 | 28 |
| **4c. Model performance for caesarean section (n total population=7587, ncases=917)** | |  | | |
| **Models** | **AUC (95% CI)** | **Sensitivity at specificity (%)** | | |
|  |  | **70%** | **80%** | **90%** |
| Core^a^ | 0.64 (0.63; 0.66) | 48 | 38 | 22 |
| **4d. Model performance for low birthweight (n total population=8340, ncases=403)** | | | | |
| **Models** | **AUC (95% CI)** | **Sensitivity at specificity (%)** | | |
|  |  | **70%** | **80%** | **90%** |
| Core^a^ | 0.66 (0.63; 0.69) | 52 | 38 | 22 |
| **4e. Model performance for macrosomia (n total population=8340, ncases=189)** | | | | |
| **Models** | **AUC (95% CI)** | **Sensitivity at specificity (%)** | | |
|  |  | **70%** | **80%** | **90%** |
| Core^a^ | 0.68 (0.64; 0.72) | 56 | 44 | 33 |

^a^Core model includes age, ethnicity, prepregnancy Body Mass Index, parity and smoking
